# Impact of Generic Alendronate Cost on the Cost-Effectiveness of Osteoporosis Screening and Treatment

**DOI:** 10.1371/journal.pone.0032879

**Published:** 2012-03-13

**Authors:** Smita Nayak, Mark S. Roberts, Susan L. Greenspan

**Affiliations:** 1 Section of Decision Sciences and Clinical Systems Modeling, Division of General Internal Medicine, Department of Medicine, University of Pittsburgh School of Medicine, Pittsburgh, Pennsylvania, United States of America; 2 Department of Health Policy and Management, University of Pittsburgh Graduate School of Public Health, Pittsburgh, Pennsylvania, United States of America; 3 Division of Endocrinology and Metabolism and Division of Geriatric Medicine, Department of Medicine, University of Pittsburgh School of Medicine, Pittsburgh, Pennsylvania, United States of America; Yale University School of Medicine, United States of America

## Abstract

**Introduction:**

Since alendronate became available in generic form in the Unites States in 2008, its price has been decreasing. The objective of this study was to investigate the impact of alendronate cost on the cost-effectiveness of osteoporosis screening and treatment in postmenopausal women.

**Methods:**

Microsimulation cost-effectiveness model of osteoporosis screening and treatment for U.S. women age 65 and older. We assumed screening initiation at age 65 with central dual-energy x-ray absorptiometry (DXA), and alendronate treatment for individuals with osteoporosis; with a comparator of “no screening” and treatment only after fracture occurrence. We evaluated annual alendronate costs of $20 through $800; outcome measures included fractures; nursing home admission; medication adverse events; death; costs; quality-adjusted life-years (QALYs); and incremental cost-effectiveness ratios (ICERs) in 2010 U.S. dollars per QALY gained. A lifetime time horizon was used, and direct costs were included. Base-case and sensitivity analyses were performed.

**Results:**

Base-case analysis results showed that at annual alendronate costs of $200 or less, osteoporosis screening followed by treatment was cost-saving, resulting in lower total costs than no screening as well as more QALYs (10.6 additional quality-adjusted life-days). When assuming alendronate costs of $400 through $800, screening and treatment resulted in greater lifetime costs than no screening but was highly cost-effective, with ICERs ranging from $714 per QALY gained through $13,902 per QALY gained. Probabilistic sensitivity analyses revealed that the cost-effectiveness of osteoporosis screening followed by alendronate treatment was robust to joint input parameter estimate variation at a willingness-to-pay threshold of $50,000/QALY at all alendronate costs evaluated.

**Conclusions:**

Osteoporosis screening followed by alendronate treatment is effective and highly cost-effective for postmenopausal women across a range of alendronate costs, and may be cost-saving at annual alendronate costs of $200 or less.

## Introduction

Osteoporosis affects approximately 10 million individuals in the United States, most of whom are postmenopausal women [1], [2]. It is estimated that half of women over the age of 50 will sustain an osteoporotic fracture during their lifetime [2], with potentially severe consequences including mortality, chronic pain, mobility limitation, and nursing home placement. Osteoporosis-related costs in the U.S. were nearly $17 billion in 2005 [3], and are projected to double or triple by 2040 [4]. The US Preventive Services Task Force (USPSTF) recommends osteoporosis screening for women aged 65 and older, to identify individuals who may be candidates for treatment [5].

Medical treatment of osteoporosis reduces fracture risk, and multiple studies have demonstrated the cost-effectiveness of osteoporosis treatment [6], [7], [8] and osteoporosis screening followed by treatment [9], [10]. Alendronate is a first-line medication for osteoporosis treatment, and is among the most cost-effective treatments for osteoporosis [6], [10], [11]. In 2008, alendronate became available in generic form in the U.S., with a resulting drop in its cost and widening of the gap in price between alendronate and other treatment options. Most published studies of the cost-effectiveness of alendronate therapy have assumed pre-2008 costs; the cost of alendronate has continued to drop since 2008; with prices currently as low as approximately $84 annually at discount pharmacies [12].

The aim of this study was to evaluate the effect of various alendronate costs on the cost-effectiveness of osteoporosis screening and treatment.

## Methods

We constructed a Monte Carlo microsimulation model of osteoporosis screening followed by alendronate treatment compared to no screening with treatment only if fracture occurs for US women age 65 and older. The model estimates direct costs in 2010 US dollars, quality-adjusted life-years (QALYs), and incremental cost-effectiveness ratios (ICERs) in units of cost per QALY gained for osteoporosis screening followed by alendronate treatment. A lifetime time horizon was used. We followed guidelines of the Panel on Cost-Effectiveness in Health and Medicine [13], and ran our analyses using TreeAge Pro Suite 2009 (TreeAge Software, Williamstown, MA). Our methods are summarized briefly here – more details can be found in a related paper in the Annals of Internal Medicine on the cost-effectiveness of different screening strategies for osteoporosis in postmenopausal women [14].

### Model Development

#### General Structure

**Figure 1:**
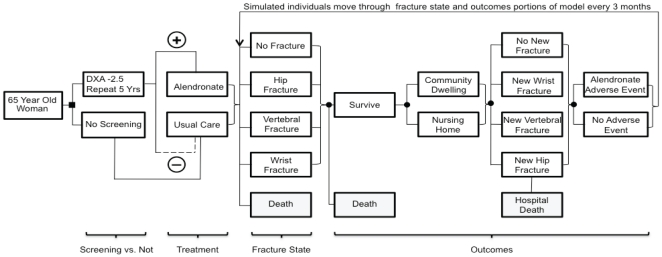
Model Schematic. A simplified and partial representation of the full model.

Figure 1 is a simplified schematic of the model, in which cohorts of 65 year old community-dwelling women are either screened with dual-energy x-ray absorptiometry (DXA) of the femoral neck and lumbar spine, or not screened and offered treatment only if an osteoporotic fracture occurs. Each woman who tests positive for osteoporosis by DXA criteria (T-score≤−2.5 at either the femoral neck or lumbar spine) is offered alendronate treatment, and each who tests negative (i.e. T-score>−2.5) receives usual care only (calcium and vitamin D). During each 3-month time period (cycle) in the model, the woman may sustain a fracture of the hip, vertebra, or wrist; may survive or die; may remain community-dwelling or enter a nursing home; and may develop an alendronate adverse event. Prior fracture history affects future fracture risk. Occurrence of a hip fractures increases the probability of nursing home placement and short-term death. Osteoporotic fractures, nursing home residence, and alendronate-related adverse events incur direct costs and “disutility” (decrease in health-related quality of life). Individuals continue cycling through the model until death. Table S1 shows model parameter assumptions.

#### Screening

We modeled initiation of screening at age 65 with DXA, with repeat screening every 5 years for individuals who test negative. With repeat screening, individuals who did not have osteoporosis at age 65 but who subsequently developed osteoporosis as their BMD declined with age would be offered treatment at the older age at which they are diagnosed. We used 65 as the screening initiation age for women in the absence of additional risk factors in accordance with current guidelines from the U.S. Preventive Services Task Force [5]. Initial DXA T-scores for each simulated individual were assigned by sampling from National Health and Nutrition Examination Survey (NHANES III) femoral neck data for non-Hispanic white women [15], and lumbar spine reference data for white women from a DXA manufacturer (Hologic, Inc., Bedford, MA). We incorporated correlations between sampled femoral neck and lumbar spine values based on published data(R = 0.603); and modeled the average annual change in T-scores at the lumbar spine and femoral neck [15], [16]. Constant, linear decrement in T-scores over time was assumed.

#### Treatment

We assumed that women with positive DXA results (T-scores≤−2.5) or who experienced a fracture of the hip, vertebra (clinically detected), or wrist were offered treatment with 70 mg of alendronate once weekly. We assumed 5 years of treatment [17], [18] and medication compliance of 50% [19], [20] in base case analysis. We assumed that the 50% of individuals who were initially compliant remained compliant with treatment for the entire duration of recommended therapy unless they sustained side effects requiring discontinuation. We assumed that 50% of individuals were entirely noncompliant with treatment recommendations, and that these individuals remained noncompliant for the entire period or recommended therapy unless they experienced an osteoporotic fracture. We assumed that previously noncompliant individuals who sustained an osteoporotic fracture had a 50% probability of becoming newly compliant. We assumed that noncompliant individuals did not incur the fracture reduction benefits or costs of alendronate therapy. All individuals, whether receiving alendronate or not, were assumed to be taking vitamin D and calcium, without additional protection against fracture.

#### Fracture Rates

Fracture rates for women not on alendronate treatment were based on Study of Osteoporotic Fractures (SOF) data, with future fracture probability predicted as a function of age, femoral neck or lumbar spine BMD, and history of fracture [14], [21]. We assumed that 35% of vertebral fractures were clinically detected [22]. For women taking alendronate, fracture relative risk was based on data from several published clinical trials and a meta-analysis [23], [24], [25], [26], [27]. For individuals who incurred a fracture, future fracture relative risk was predicted using data for women with previous fractures [27]. For women receiving alendronate treatment without a history of osteoporotic fracture, we based future fracture relative risk on data for women without previous fractures [23]–[26], using the fracture risk estimates corresponding to the lower of the T-scores from the lumbar spine or femoral neck.

We assumed a constant, linear decline in fracture risk reduction benefit over 5 years after completion of alendronate treatment [28].

#### Mortality Rates

We used US national vital statistics data for baseline mortality rates [29]; and data on hip fracture-related mortality from several published sources [30], [31].

#### Nursing Home Characteristics

Nursing home admission rates, length of stay, and mortality data were obtained from several published sources [31], [32], [33], [34].

#### Costs

We included direct costs of DXA screening ($97.71) [35], alendronate therapy, fracture treatment, physician visits, nursing home residence, and alendronate-related adverse events. In separate base-case analyses, we evaluated annual alendronate costs of $20, $40, $60, $80, $100, $200, $400, $600, and $800. These costs were chosen to represent a spectrum of possible alendronate annual costs, including a cost higher than the 2010 CMS Federal Upper Limit price for alendronate (approximately $738) and costs lower than the current annual cost of alendronate at discount pharmacies (approximately $84 ) [12]. Costs for fracture-related treatment, other relevant medical services, and nursing home stay were obtained from several sources [35]–[37]. We inflated costs to 2010 U.S. dollars using the US Consumer Price Index for Medical Care [38]. We discounted future costs by 3% annually.

#### Utilities

We used data from a sample of older women in the U.S. for baseline health state utilites [39]. We modeled disutility from osteoporotic fractures, nursing home placement, and alendronate adverse events using data from multiple published sources [40], [41], [42], [43], [44], [45], [46]. We discounted future utility values by 3% annually.

#### Adverse Events

We modeled medication adverse events of esophagitis and esophageal ulceration with rates obtained from clinical trials data [27].

### Analyses

We performed base-case and sensitivity analyses separately for each alendronate cost evaluated. Key parameter values used for base-case analyses and the range of values used for sensitivity analyses are shown in Table S1. Sensitivity analyses included evaluation of different assumptions for key model parameters of costs (higher than base-case); discount rate (5% annually instead of 3%); fracture risks (50% lower than base-case); and probability of admission to a nursing home after hip fracture (30% instead of 60%). Additionally, probabilistic sensitivity analyses were performed to evaluate the impact of joint input parameter uncertainty on the model findings. For each base-case analysis and for the sensitivity analyses of costs, discount rate, fracture risks, and probability of nursing home admission, we ran the model with 1 million trials. For each probabilistic sensitivity analysis, we ran 500 simulations with 2,000 trials per simulation.

### Model Validation

We compared the model's fracture and life expectancy predictions with published U.S. outcomes data.

## Results

### Model Validation

Our model predicted a mean life expectancy of 19.3 years for 65-year-old women who were not screened for osteoporosis. This is similar to the U.S. National Vital Statistics figure of 19.8 years reported for 65-year-old women in 2006 [47]. The model predicted that 49% of 65-year-old women who were not screened would experience at least one osteoporotic fracture during their lifetime. Our model predicted that 28% of women would experience a vertebral fracture; this figure matches that reported in a prior study of older US women [48]. Our model predicted that 24% of women would sustain a hip fracture during their lifetime, and 17% would sustain a wrist fracture; these estimates are higher than those reported in a study of Medicare beneficiaries who sustained a fracture by age 90, which used data from 1986–1990 [49]. However, women's life expectancies have increased by 1.3 years since 1988, and 29% of women lived to be at least 90 years old in our modeling analysis; 17% of women in our analysis experienced a hip fracture before age 90, close to the figure reported by Barrett et al. [49].

### Base-Case Analyses

Osteoporosis screening initiated at age 65 followed by alendronate treatment was more effective than no screening with treatment only if fracture occurs, resulting in 10.6 additional quality-adjusted life-days. When assuming alendronate costs of $200 or less, osteoporosis screening and treatment was cost-saving, resulting in lower total costs than no screening as well as more QALYs (Table 1). Lifetime direct cost savings ranged from $171 to $343 when assuming alendronate annual costs of $200 or $20, respectively. When assuming alendronate costs of $400, $600, or $800, screening and treatment resulted in greater lifetime costs than no screening but was highly cost-effective, with ICERs ranging from $714 per QALY gained to $13,902 per QALY gained when assuming annual alendronate costs of $400 or $800, respectively (Table 1).

**Table 1:** Base-Case Analysis Results, Various Alendronate Costs.

Alendronate Cost ($)	Incremental Cost-Effectiveness Ratio ($/QALY)^a^
20	Cost-saving: $343^b^
40	Cost-saving: $324^b^
60	Cost-saving: $305^b^
80	Cost-saving: $286^b^
100	Cost-saving: $266^b^
200	Cost-saving: $171^b^
400	$712
600	$7307
800	$13,902

a Incremental cost-effectiveness of osteoporosis screening followed by alendronate treatment, compared to no screening with treatment only if fracture occurs; in 2010 US dollars per quality-adjusted life-year (QALY).

b Lifetime direct costs saved.

### Sensitivity Analyses

Table 2 shows results (ICERs and cost savings) from sensitivity analyses of assumptions for costs, discount rate, fracture risks, and probability of nursing home admission after hip fracture. In general, these results were similar to base-case analysis findings in demonstrating the value of osteoporosis screening followed by alendronate treatment across a range of alendronate annual costs. However, ICERs or cost savings associated with different alendronate costs varied. When assuming 50% lower fracture risks, screening and treatment remained cost-effective across the range of costs evaluated, but with higher ICERs than in base-case analysis; additionally, none of the alendronate costs evaluated were associated with cost savings. When assuming nursing home admission probability of 30% after hip fracture instead of 60%, screening and treatment remained highly cost-effective, but with ICERs higher than in base-case analysis; additionally, the annual cost at which alendronate became cost-saving was $40 instead of $200. When assuming high costs for fracture-related treatment, nursing home care, and DXA, screening and treatment remained highly cost-effective, but with ICERs higher than in base-case analysis; additionally, the cost at which alendronate became cost-saving was $100 instead of $200. When assuming a discount rate of 5% instead of 3%, results were similar to base-case analysis, with screening and treatment becoming cost-saving at annual alendronate costs of $200 or lower.

**Table 2:** Sensitivity Analysis Results; Costs, Discount Rate, Fracture Risk, Nursing Home Probability.

Alendronate Cost ($)	Incremental Cost-Effectiveness Ratio ($/QALY)^a^			
	High Costs Scenario^b^	High Discount Rate Scenario^c^	Low Fracture Risk Scenario^d^	Low Nursing Home Probability Scenario^e^
20	Cost-saving: $116^f^	Cost-saving: $275^f^	$5483	Cost-saving: $28^f^
40	Cost-saving: $97^f^	Cost-saving: $258^f^	$6728	Cost-saving: $9^f^
60	Cost-saving: $77^f^	Cost-saving: $241^f^	$7973	$362
80	Cost-saving: $58^f^	Cost-saving: $223^f^	$9218	$1047
100	Cost-saving: $39^f^	Cost-saving: $206^f^	$10463	$1733
200	$1948	Cost-saving: $119^f^	$16688	$5161
400	$8543	$2561	$29138	$12018
600	$15138	$10638	$41588	$18874
800	$21733	$18715	$54037	$25731

a Incremental cost-effectiveness of osteoporosis screening followed by alendronate treatment, compared to no screening with treatment only if fracture occurs; in 2010 US dollars per quality-adjusted life-year (QALY).

b High fracture-related, nursing home, and dual-energy x-ray absorptiometry costs (high values of the sensitivity analysis range for costs shown in Table S1).

c Discount rate for future costs and health state utilities of 5% annually.

d Fracture risks (hip, vertebral, and wrist) 50% lower than in base-case analysis.

e Probability of nursing home admission after hip fracture of 30%.

f Lifetime direct costs saved.

**Figure 2:**
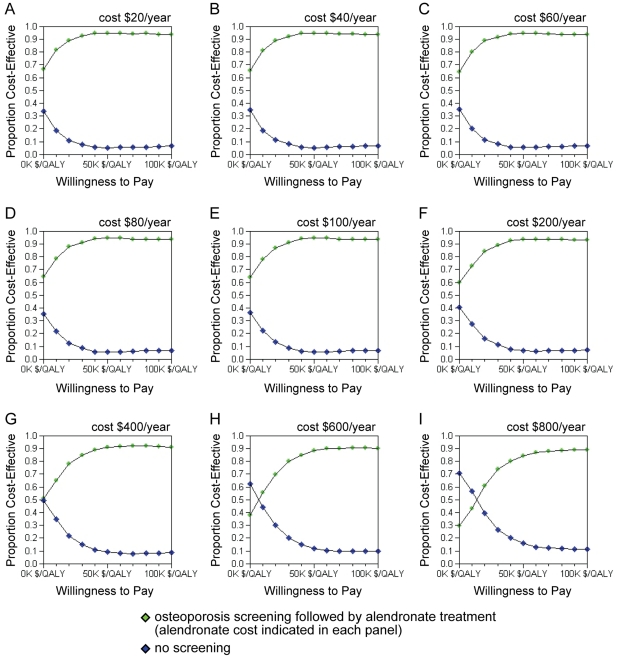
Probabilistic Sensitivity Analysis Cost-Effectiveness Acceptability Curves.

Probabilistic sensitivity analyses revealed that the cost-effectiveness of osteoporosis screening followed by alendronate treatment was relatively robust to variations in input parameter estimates at a willingness-to-pay threshold of $50,000/QALY for all alendronate costs evaluated (Figure 2). When assuming annual alendronate costs of $100 or less, the probability that osteoporosis screening followed by alendronate treatment was cost-effective was 95%. The probability that screening followed by alendronate treatment was cost effective remained high at 84% when assuming an annual alendronate cost of $800.

## Discussion

Our analyses demonstrated that osteoporosis screening followed by alendronate treatment is effective and highly cost-effective for women aged 65 and older across a wide range of alendronate costs; and potentially cost-saving at annual alendronate costs of $200 or less, depending on assumptions about fracture risk, nursing home admission probability after hip fracture, and health care costs. Sensitivity analyses showed that the value (cost-effectiveness) of alendronate treatment for all alendronate costs evaluated was relatively robust to key model parameter uncertainty; but the price at which alendronate becomes cost-saving is sensitive to the parameters specified above. These results indicate that osteoporosis screening followed by alendronate treatment is an advantageous use of healthcare resources for women age 65 and older, that can be expected to improve health outcomes and may result in potential cost savings when alendronate annual costs are $200 or less, as is currently the case at discount pharmacies. As the cost of alendronate continues to fall, the value and potential for cost savings for the U.S. healthcare system resulting from osteoporosis screening of older women followed by alendronate treatment can be expected to increase, assuming appropriate selection of candidates for treatment. This is a significant finding, given how few preventive services can result in cost savings [50], [51].

Our model has several limitations. First, our analyses did not incorporate the costs of added life days from osteoporosis screening. However, the costs of added life days would likely be small as the age of death was very similar in the screening followed by treatment and no screening model arms. Second, our analysis assumed that only women with DXA T-scores in the osteoporotic range would be offered treatment, in accordance with evidence that treatment of women with osteopenia (low bone mass) is not cost-effective [52]. However, if screening leads to inappropriate treatment of individuals at lower risk for osteoporotic fracture, it's cost-effectiveness and potential cost savings would be lessened. Additionally, we did not model all potential adverse events of alendronate treatment, including arthalgias, myalgias, osteonecrosis of the jaw, or atypical femoral neck fractures; such adverse events may require additional physician visits, labwork, or discontinuation of medication. However, osteonecrosis of the jaw and atypical femoral neck fractures are rare reported adverse events, and their association with alendronate treatment is still under investigation. Moreover, the effectiveness and adherence with generic bisphosphonate therapy may be lower than with proprietary formulations [53]. If this is the case, and adherence or fracture risk reduction with generic alendronate is lower than our model assumptions, generic alendronate therapy would be less cost-effective than our findings suggest. Finally, our model parameter inputs were primarily based on data from white women, and thus our results may be less applicable to women of other races.

In conclusion, our analyses indicate that osteoporosis screening followed by alendronate treatment is highly cost-effective for women aged 65 and older when assuming annual alendronate costs of $400 through $800, and potentially cost-saving when assuming annual alendronate costs of $200 or less, depending on key parameter assumptions. Thus, osteoporosis screening followed by alendronate treatment in appropriately selected patients represents an excellent healthcare value, and this important preventive health service should be promoted. Although our analyses were limited to alendronate, other osteoporosis treatments with similar effectiveness and costs may be expected to be similarly cost-effective. For example, other available oral bisphosphonates (e.g. risedronate) may be similarly cost-effective if costs of the medication were to decrease. However intravenous bisphosphonates, which have additional costs for the infusions, different adverse event profiles, as well as different fracture reduction outcomes would not be expected to have similar cost-effectiveness to alendronate. This would apply to other osteoporosis medications that have different costs, adverse event profiles, adherence patterns, and routes of administration.

Future research should evaluate the cost-effectiveness of “real-world” osteoporosis screening and treatment practices, in which some patients will be inappropriately selected for treatment. Furthermore the effects of assuming treatment duration longer than 5 years or a drug holiday should be examined. However, assuming appropriate selection of individuals for treatment, osteoporosis screening followed by alendronate treatment in women aged 65 and old represents a superb healthcare value across the variety of alendronate costs evaluated.

## Supporting Information

Table S1 **Key Model Parameter Assumptions.**
(DOC)
