# Supplementary material for: Impact of Generic Alendronate Cost on the Cost-Effectiveness of Osteoporosis Screening and Treatment
Source: PLoS One. 2012 Mar 13;7(3):e32879. doi: 10.1371/journal.pone.0032879 (PMC3302782; doi:10.1371/journal.pone.0032879)
Supplement: Table S1 — Key Model Parameter Assumptions. (DOC) [file pone.0032879.s001.doc]

**Table S1. Key Model Parameter Assumptions**

| **Parameter** | **Base-Case Value** | **Probabilistic Sensitivity Analysis Values (Range)**^a^ | **Data Sources** |
| --- | --- | --- | --- |
| **Costs, 2010 US dollars**^b^ | | | |
| Nursing home care, annual cost | 74,846 | 60,000-90,000^c^ | GE Financial Nursing Home Cost of Care Survey [37] (base-case value) |
| Hip fracture, direct medical costs | 22,528 | 11,264-33,792^d^ | Gabriel et al [36] (base-case value) |
| Clinical vertebral fracture, direct medical costs | 9,214 | 4,607-13,821^d^ | Gabriel et al [36] (base-case value) |
| Wrist fracture, direct medical costs | 5,003 | 2,502-7,505^d^ | Gabriel et al [36] (base-case value) |
| Central dual-energy x-ray absorptiometry (DXA) | 97.71 | 60-120^c^ | Centers for Medicare and Medicaid Services [35] (base-case value) |
| Physician visit (CPT code 99213) | 66.74 | N/A | Centers for Medicare and Medicaid Services [35] (base-case value) |
| **Health state utility values** | | | |
| No fracture, age 65 years | 0.811 | 0.730-0.892^e^ | Hanmer et al [39] (base-case value) |
| No fracture, age 75 years | 0.771 | 0.694-0.848^e^ | Hanmer et al [39] (base-case value) |
| No fracture, age 85 years | 0.724 | 0.652-0.796^e^ | Hanmer et al [39] (base-case value) |
| Nursing home placement (multiplier) | 0.4 | 0.2-0.6^c^ | Brazier et al [40] (base-case value) |
| Hip fracture, first year/subsequent years (multiplier) | 0.797/0.9 | 0.717-0.877/0.81-0.99^e^ | Brazier et al [44] (first year multiplier); Brazier et al [40] (subsequent years multiplier) |
| Vertebral fracture, first year/subsequent years (multiplier) | 0.82/0.931 | 0.740-0.902/0.840-1.0^e^ | Kanis et al [45] (base-case values) |
| Wrist fracture, first year/subsequent years (multiplier) | 0.981/1.0 | 0.95-1.0/1.0^c^ | Dolan et al [42] (first year multiplier); Brazier et al [40] (subsequent years multiplier) |
| Esophagitis (multiplier)^f^ | 0.98 | N/A | Fryback et al [43] (base-case value)^f^ |
| Esophageal ulcer (multiplier)^f^ | 0.91 | N/A | Fryback et al [43] (base-case value)^f^ |
| **Relative risk of fracture on alendronate treatment** | | | |
| Hip fracture (history of prior vertebral fracture) | 0.49 | 0.34-0.64^g^ | Black et al [27] |
| Hip fracture (femoral neck T-score –2.5 or less) | 0.44 | 0.31-0.57^g^ | Cummings et al [25] |
| Hip fracture (lumbar spine T-score –2.5 or less) | 0.46 | 0.32-0.60^g^ | Karpf et al [24] |
| Vertebral fracture (history of prior vertebral fracture) | 0.53 | 0.37-0.69^g^ | Black et al [27] |
| Vertebral fracture (femoral neck T-score –2.5 or less) | 0.50 | 0.35-0.65^g^ | Cummings et al [25] |
| Vertebral fracture (lumbar spine T-score –2.5 or less) | 0.52 | 0.36-0.68^g^ | Liberman et al [23] |
| Vertebral fracture (femoral neck or lumbar spine T-score –2.0 or less and greater than –2.5) | 0.54 | 0.38-0.70^g^ | Cummings et al [25] |
| Vertebral fracture (femoral neck or lumbar spine T-score –1.5 or less and greater than –2.0) | 0.82 | 0.57-1.07^g^ | Cummings et al [25] |
| Wrist fracture (history of prior vertebral fracture) | 0.52 | 0.36-0.68^g^ | Black et al [27] |
| Wrist fracture (femoral neck T-score –2.5 or less) | 0.88 | 0.62-1.14^g^ | Cummings et al [25] |
| Wrist fracture (lumbar spine T-score –2.5 or less) | 0.39 | 0.27-0.51^g^ | Karpf et al [24] |
| Non-vertebral fracture (femoral neck or lumbar spine T-score greater than –2.5) | 1.0 | N/A | Cummings et al [25] |
| **Admission to nursing home after hip fracture** | | | |
| Rate of admission | 0.60 | 0.42-0.78^g^ | Braithwaite et al [33], Fitzgerald et al [34] (base-case value) |
| **Alendronate treatment** | | | |
| Compliance with treatment, % | 50 | 30-70^c^ | Solomon et al [19] (base-case value) |
| Length of treatment, years | 5 | N/A | Black et al [17]; Schwartz et al [18] |
| **Discount rate** | | | |
| Costs | 0.03 | N/A | Assumed |
| Quality-adjusted life-years | 0.03 | N/A | Assumed |

Abbreviations: CPT, current procedural terminology; N/A, not applicable

^a^ Triangular probability distributions used.

^b^ Costs not presented in 2010 dollars were inflated to 2010 dollars using the Consumer Price Index for Medical Care.

^c^ Sensitivity analysis values assumed.

^d^ Sensitivity analysis values 50% lower and 50% higher than base-case value.

^e^ Sensitivity analysis values 10% lower and 10% higher than base-case value.

^f^ Surrogate utility value used.

^g^ Sensitivity analysis values 30% lower and 30% higher than base-case value.
